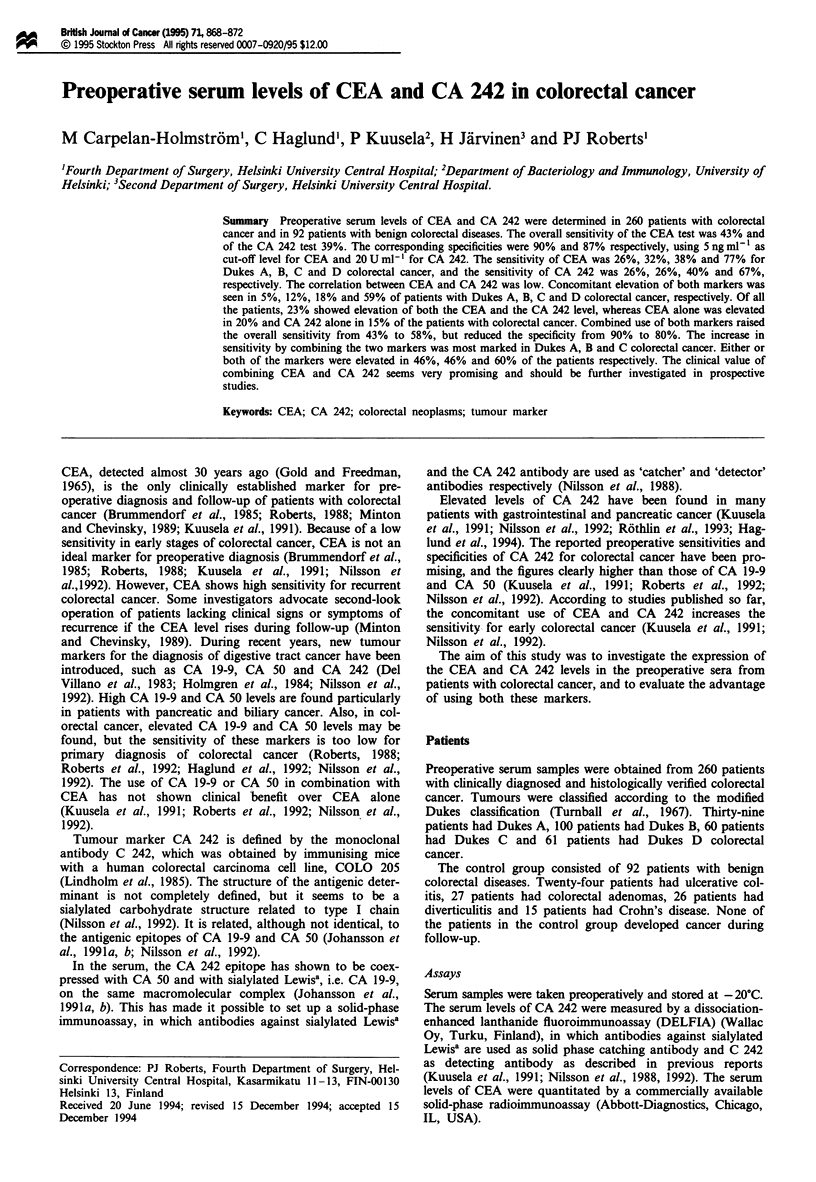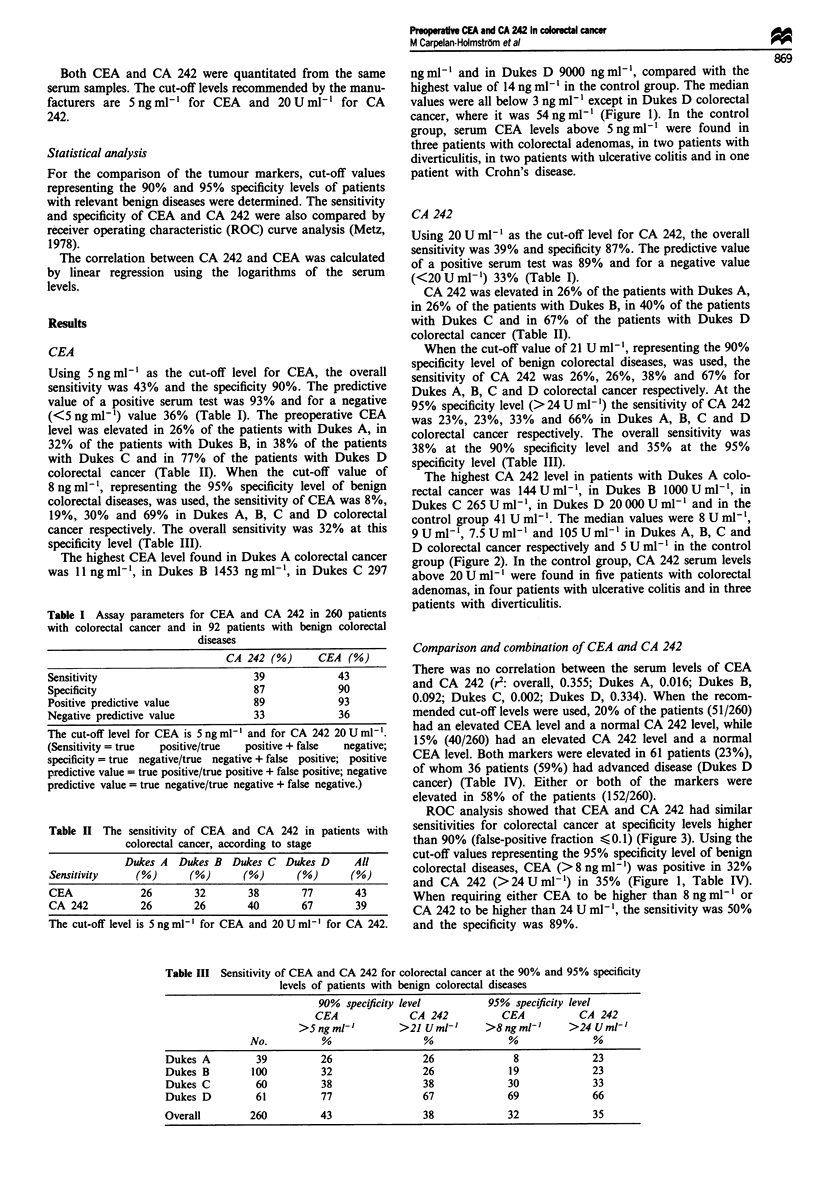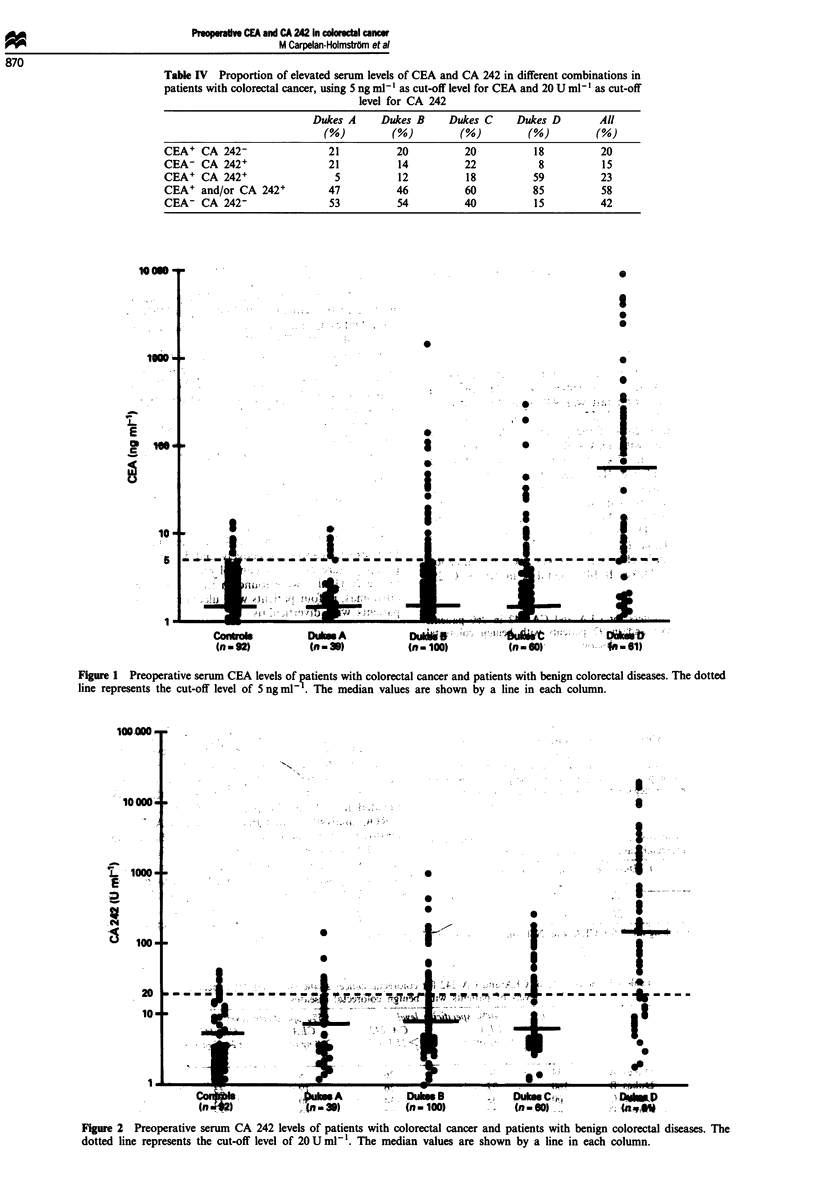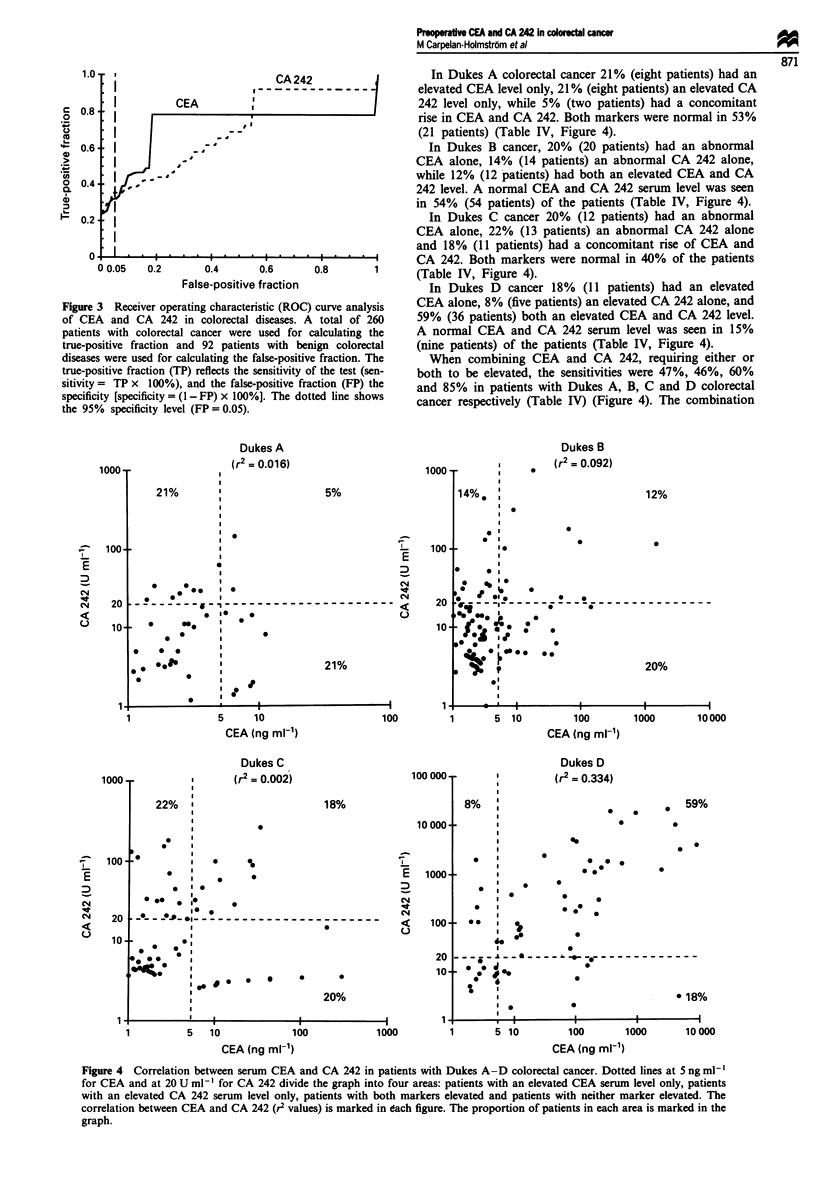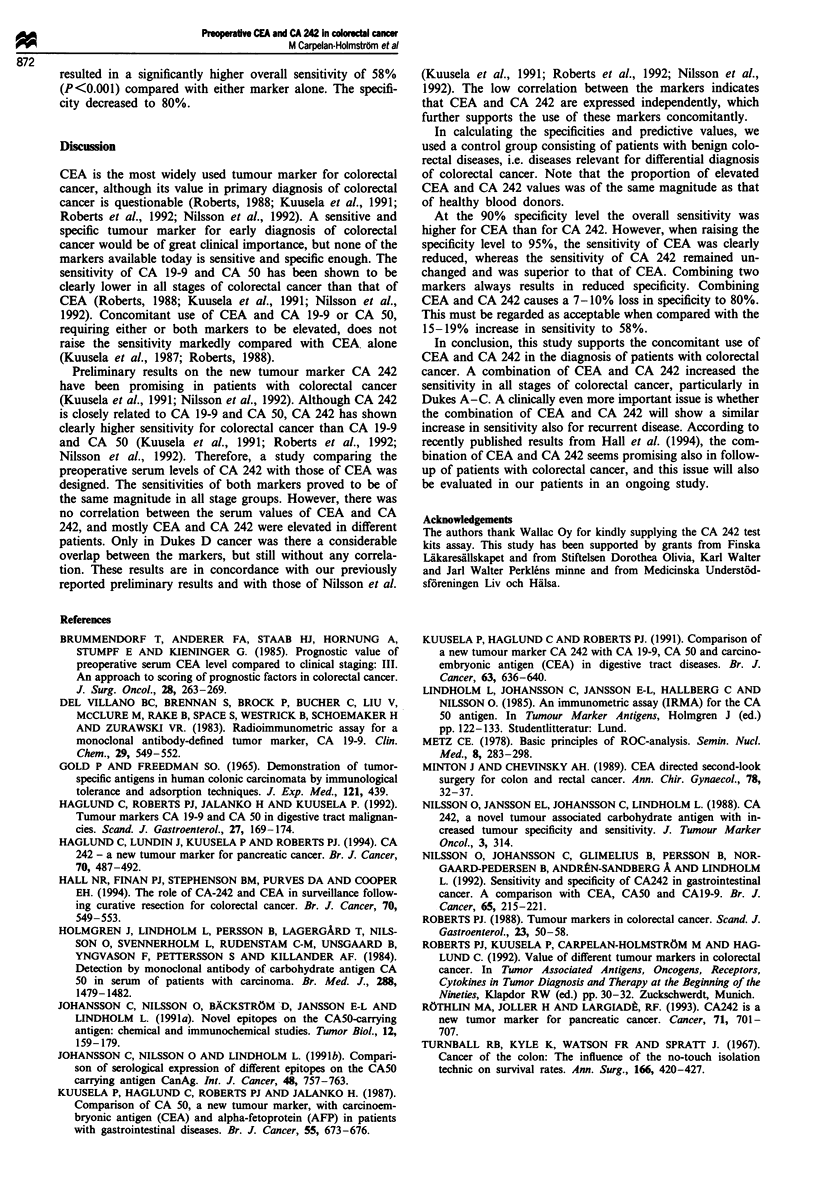# Preoperative serum levels of CEA and CA 242 in colorectal cancer.

**DOI:** 10.1038/bjc.1995.167

**Published:** 1995-04

**Authors:** M. Carpelan-Holmström, C. Haglund, P. Kuusela, H. Järvinen, P. J. Roberts

**Affiliations:** Fourth Department of Surgery, Helsinki University Central Hospital, Finland.

## Abstract

Preoperative serum levels of CEA and CA 242 were determined in 260 patients with colorectal cancer and in 92 patients with benign colorectal diseases. The overall sensitivity of the CEA test was 43% and of the CA 242 test 39%. The corresponding specificities were 90% and 87% respectively, using 5 ng ml-1 as cut-off level for CEA and 20 U ml-1 for CA 242. The sensitivity of CEA was 26%, 32%, 38% and 77% for Dukes A, B, C and D colorectal cancer, and the sensitivity of CA 242 was 26%, 26%, 40% and 67%, respectively. The correlation between CEA and CA 242 was low. Concomitant elevation of both markers was seen in 5%, 12%, 18% and 59% of patients with Dukes A, B, C and D colorectal cancer, respectively. Of all the patients, 23% showed elevation of both the CEA and the CA 242 level, whereas CEA alone was elevated in 20% and CA 242 alone in 15% of the patients with colorectal cancer. Combined use of both markers raised the overall sensitivity from 43% to 58%, but reduced the specificity from 90% to 80%. The increase in sensitivity by combining the two markers was most marked in Dukes A, B and C colorectal cancer. Either or both of the markers were elevated in 46%, 46% and 60% of the patients respectively. The clinical value of combining CEA and CA 242 seems very promising and should be further investigated in prospective studies.


British Journal of Cancer (1995) 71, 868-872
© 1995 Stockton Press All rights reserved 0007-0920/95 $12.00

### Preoperative serum levels of CEA and CA 242 in colorectal cancer

M Carpelan-Holmström[1], C Haglund[1], P Kuusela[2], H Järvinen[3] and PJ Roberts[1]

[1]*Fourth Department of Surgery, Helsinki University Central Hospital;* [2]*Department of Bacteriology and Immunology, University of Helsinki;* [3]*Second Department of Surgery, Helsinki University Central Hospital.*

**Summary** Preoperative serum levels of CEA and CA 242 were determined in 260 patients with colorectal cancer and in 92 patients with benign colorectal diseases. The overall sensitivity of the CEA test was 43% and of the CA 242 test 39%. The corresponding specificities were 90% and 87% respectively, using 5 ng ml$^{-1}$ as cut-off level for CEA and 20 U ml$^{-1}$ for CA 242. The sensitivity of CEA was 26%, 32%, 38% and 77% for Dukes A, B, C and D colorectal cancer, and the sensitivity of CA 242 was 26%, 26%, 40% and 67%, respectively. The correlation between CEA and CA 242 was low. Concomitant elevation of both markers was seen in 5%, 12%, 18% and 59% of patients with Dukes A, B, C and D colorectal cancer, respectively. Of all the patients, 23% showed elevation of both the CEA and the CA 242 level, whereas CEA alone was elevated in 20% and CA 242 alone in 15% of the patients with colorectal cancer. Combined use of both markers raised the overall sensitivity from 43% to 58%, but reduced the specificity from 90% to 80%. The increase in sensitivity by combining the two markers was most marked in Dukes A, B and C colorectal cancer. Either or both of the markers were elevated in 46%, 46% and 60% of the patients respectively. The clinical value of combining CEA and CA 242 seems very promising and should be further investigated in prospective studies.

**Keywords:** CEA; CA 242; colorectal neoplasms; tumour marker

CEA, detected almost 30 years ago (Gold and Freedman, 1965), is the only clinically established marker for preoperative diagnosis and follow-up of patients with colorectal cancer (Brummendorf *et al.*, 1985; Roberts, 1988; Minton and Chevinsky, 1989; Kuusela *et al.*, 1991). Because of a low sensitivity in early stages of colorectal cancer, CEA is not an ideal marker for preoperative diagnosis (Brummendorf *et al.*, 1985; Roberts, 1988; Kuusela *et al.*, 1991; Nilsson *et al.*,1992). However, CEA shows high sensitivity for recurrent colorectal cancer. Some investigators advocate second-look operation of patients lacking clinical signs or symptoms of recurrence if the CEA level rises during follow-up (Minton and Chevinsky, 1989). During recent years, new tumour markers for the diagnosis of digestive tract cancer have been introduced, such as CA 19-9, CA 50 and CA 242 (Del Villano *et al.*, 1983; Holmgren *et al.*, 1984; Nilsson *et al.*, 1992). High CA 19-9 and CA 50 levels are found particularly in patients with pancreatic and biliary cancer. Also, in colorectal cancer, elevated CA 19-9 and CA 50 levels may be found, but the sensitivity of these markers is too low for primary diagnosis of colorectal cancer (Roberts, 1988; Roberts *et al.*, 1992; Haglund *et al.*, 1992; Nilsson *et al.*, 1992). The use of CA 19-9 or CA 50 in combination with CEA has not shown clinical benefit over CEA alone (Kuusela *et al.*, 1991; Roberts *et al.*, 1992; Nilsson *et al.*, 1992).

Tumour marker CA 242 is defined by the monoclonal antibody C 242, which was obtained by immunising mice with a human colorectal carcinoma cell line, COLO 205 (Lindholm *et al.*, 1985). The structure of the antigenic determinant is not completely defined, but it seems to be a sialylated carbohydrate structure related to type I chain (Nilsson *et al.*, 1992). It is related, although not identical, to the antigenic epitopes of CA 19-9 and CA 50 (Johansson *et al.*, 1991*a*, *b*; Nilsson *et al.*, 1992).

In the serum, the CA 242 epitope has shown to be coexpressed with CA 50 and with sialylated Lewis[a], i.e. CA 19-9, on the same macromolecular complex (Johansson *et al.*, 1991*a*, *b*). This has made it possible to set up a solid-phase immunoassay, in which antibodies against sialylated Lewis[a] and the CA 242 antibody are used as 'catcher' and 'detector' antibodies respectively (Nilsson *et al.*, 1988).

Elevated levels of CA 242 have been found in many patients with gastrointestinal and pancreatic cancer (Kuusela *et al.*, 1991; Nilsson *et al.*, 1992; Röthlin *et al.*, 1993; Haglund *et al.*, 1994). The reported preoperative sensitivities and specificities of CA 242 for colorectal cancer have been promising, and the figures clearly higher than those of CA 19-9 and CA 50 (Kuusela *et al.*, 1991; Roberts *et al.*, 1992; Nilsson *et al.*, 1992). According to studies published so far, the concomitant use of CEA and CA 242 increases the sensitivity for early colorectal cancer (Kuusela *et al.*, 1991; Nilsson *et al.*, 1992).

The aim of this study was to investigate the expression of the CEA and CA 242 levels in the preoperative sera from patients with colorectal cancer, and to evaluate the advantage of using both these markers.

### Patients

Preoperative serum samples were obtained from 260 patients with clinically diagnosed and histologically verified colorectal cancer. Tumours were classified according to the modified Dukes classification (Turnball *et al.*, 1967). Thirty-nine patients had Dukes A, 100 patients had Dukes B, 60 patients had Dukes C and 61 patients had Dukes D colorectal cancer.

The control group consisted of 92 patients with benign colorectal diseases. Twenty-four patients had ulcerative colitis, 27 patients had colorectal adenomas, 26 patients had diverticulitis and 15 patients had Crohn's disease. None of the patients in the control group developed cancer during follow-up.

### *Assays*

Serum samples were taken preoperatively and stored at −20°C. The serum levels of CA 242 were measured by a dissociation-enhanced lanthanide fluoroimmunoassay (DELFIA) (Wallac Oy, Turku, Finland), in which antibodies against sialylated Lewis[a] are used as solid phase catching antibody and C 242 as detecting antibody as described in previous reports (Kuusela *et al.*, 1991; Nilsson *et al.*, 1988, 1992). The serum levels of CEA were quantitated by a commercially available solid-phase radioimmunoassay (Abbott-Diagnostics, Chicago, IL, USA).

Correspondence: PJ Roberts, Fourth Department of Surgery, Helsinki University Central Hospital, Kasarmikatu 11-13, FIN-00130 Helsinki 13, Finland
Received 20 June 1994; revised 15 December 1994; accepted 15 December 1994

Both CEA and CA 242 were quantitated from the same serum samples. The cut-off levels recommended by the manufacturers are 5 ng $ml^{-1}$ for CEA and 20 U $ml^{-1}$ for CA 242.

*Statistical analysis*

For the comparison of the tumour markers, cut-off values representing the 90% and 95% specificity levels of patients with relevant benign diseases were determined. The sensitivity and specificity of CEA and CA 242 were also compared by receiver operating characteristic (ROC) curve analysis (Metz, 1978).

The correlation between CA 242 and CEA was calculated by linear regression using the logarithms of the serum levels.

### Results

*CEA*

Using 5 ng $ml^{-1}$ as the cut-off level for CEA, the overall sensitivity was 43% and the specificity 90%. The predictive value of a positive serum test was 93% and for a negative (<5 ng $ml^{-1}$) value 36% (Table I). The preoperative CEA level was elevated in 26% of the patients with Dukes A, in 32% of the patients with Dukes B, in 38% of the patients with Dukes C and in 77% of the patients with Dukes D colorectal cancer (Table II). When the cut-off value of 8 ng $ml^{-1}$, representing the 95% specificity level of benign colorectal diseases, was used, the sensitivity of CEA was 8%, 19%, 30% and 69% in Dukes A, B, C and D colorectal cancer respectively. The overall sensitivity was 32% at this specificity level (Table III).

The highest CEA level found in Dukes A colorectal cancer was 11 ng $ml^{-1}$, in Dukes B 1453 ng $ml^{-1}$, in Dukes C 297 ng $ml^{-1}$ and in Dukes D 9000 ng $ml^{-1}$, compared with the highest value of 14 ng $ml^{-1}$ in the control group. The median values were all below 3 ng $ml^{-1}$ except in Dukes D colorectal cancer, where it was 54 ng $ml^{-1}$ (Figure 1). In the control group, serum CEA levels above 5 ng $ml^{-1}$ were found in three patients with colorectal adenomas, in two patients with diverticulitis, in two patients with ulcerative colitis and in one patient with Crohn's disease.

**Table I** Assay parameters for CEA and CA 242 in 260 patients with colorectal cancer and in 92 patients with benign colorectal diseases

| | *CA 242 (%)* | *CEA (%)* |
|---|---|---|
| Sensitivity | 39 | 43 |
| Specificity | 87 | 90 |
| Positive predictive value | 89 | 93 |
| Negative predictive value | 33 | 36 |

The cut-off level for CEA is 5 ng $ml^{-1}$ and for CA 242 20 U $ml^{-1}$. (Sensitivity = true positive/true positive + false negative; specificity = true negative/true negative + false positive; positive predictive value = true positive/true positive + false positive; negative predictive value = true negative/true negative + false negative.)

**Table II** The sensitivity of CEA and CA 242 in patients with colorectal cancer, according to stage

| *Sensitivity* | *Dukes A (%)* | *Dukes B (%)* | *Dukes C (%)* | *Dukes D (%)* | *All (%)* |
|---|---|---|---|---|---|
| CEA | 26 | 32 | 38 | 77 | 43 |
| CA 242 | 26 | 26 | 40 | 67 | 39 |

The cut-off level is 5 ng $ml^{-1}$ for CEA and 20 U $ml^{-1}$ for CA 242.

*CA 242*

Using 20 U $ml^{-1}$ as the cut-off level for CA 242, the overall sensitivity was 39% and specificity 87%. The predictive value of a positive serum test was 89% and for a negative value (<20 U $ml^{-1}$) 33% (Table I).

CA 242 was elevated in 26% of the patients with Dukes A, in 26% of the patients with Dukes B, in 40% of the patients with Dukes C and in 67% of the patients with Dukes D colorectal cancer (Table II).

When the cut-off value of 21 U $ml^{-1}$, representing the 90% specificity level of benign colorectal diseases, was used, the sensitivity of CA 242 was 26%, 26%, 38% and 67% for Dukes A, B, C and D colorectal cancer respectively. At the 95% specificity level (>24 U $ml^{-1}$) the sensitivity of CA 242 was 23%, 23%, 33% and 66% in Dukes A, B, C and D colorectal cancer respectively. The overall sensitivity was 38% at the 90% specificity level and 35% at the 95% specificity level (Table III).

The highest CA 242 level in patients with Dukes A colorectal cancer was 144 U $ml^{-1}$, in Dukes B 1000 U $ml^{-1}$, in Dukes C 265 U $ml^{-1}$, in Dukes D 20 000 U $ml^{-1}$ and in the control group 41 U $ml^{-1}$. The median values were 8 U $ml^{-1}$, 9 U $ml^{-1}$, 7.5 U $ml^{-1}$ and 105 U $ml^{-1}$ in Dukes A, B, C and D colorectal cancer respectively and 5 U $ml^{-1}$ in the control group (Figure 2). In the control group, CA 242 serum levels above 20 U $ml^{-1}$ were found in five patients with colorectal adenomas, in four patients with ulcerative colitis and in three patients with diverticulitis.

*Comparison and combination of CEA and CA 242*

There was no correlation between the serum levels of CEA and CA 242 ($r^2$: overall, 0.355; Dukes A, 0.016; Dukes B, 0.092; Dukes C, 0.002; Dukes D, 0.334). When the recommended cut-off levels were used, 20% of the patients (51/260) had an elevated CEA level and a normal CA 242 level, while 15% (40/260) had an elevated CA 242 level and a normal CEA level. Both markers were elevated in 61 patients (23%), of whom 36 patients (59%) had advanced disease (Dukes D cancer) (Table IV). Either or both of the markers were elevated in 58% of the patients (152/260).

ROC analysis showed that CEA and CA 242 had similar sensitivities for colorectal cancer at specificity levels higher than 90% (false-positive fraction ⩽0.1) (Figure 3). Using the cut-off values representing the 95% specificity level of benign colorectal diseases, CEA (>8 ng $ml^{-1}$) was positive in 32% and CA 242 (>24 U $ml^{-1}$) in 35% (Figure 1, Table IV). When requiring either CEA to be higher than 8 ng $ml^{-1}$ or CA 242 to be higher than 24 U $ml^{-1}$, the sensitivity was 50% and the specificity was 89%.

**Table III** Sensitivity of CEA and CA 242 for colorectal cancer at the 90% and 95% specificity levels of patients with benign colorectal diseases

| | | *90% specificity level* | | *95% specificity level* | |
|---|---|---|---|---|---|
| | *No.* | *CEA >5 ng $ml^{-1}$ %* | *CA 242 >21 U $ml^{-1}$ %* | *CEA >8 ng $ml^{-1}$ %* | *CA 242 >24 U $ml^{-1}$ %* |
| Dukes A | 39 | 26 | 26 | 8 | 23 |
| Dukes B | 100 | 32 | 26 | 19 | 23 |
| Dukes C | 60 | 38 | 38 | 30 | 33 |
| Dukes D | 61 | 77 | 67 | 69 | 66 |
| Overall | 260 | 43 | 38 | 32 | 35 |

**Table IV** Proportion of elevated serum levels of CEA and CA 242 in different combinations in patients with colorectal cancer, using 5 ng ml$^{-1}$ as cut-off level for CEA and 20 U ml$^{-1}$ as cut-off level for CA 242

| | *Dukes A (%)* | *Dukes B (%)* | *Dukes C (%)* | *Dukes D (%)* | *All (%)* |
|---|---|---|---|---|---|
| CEA$^+$ CA 242$^-$ | 21 | 20 | 20 | 18 | 20 |
| CEA$^-$ CA 242$^+$ | 21 | 14 | 22 | 8 | 15 |
| CEA$^+$ CA 242$^+$ | 5 | 12 | 18 | 59 | 23 |
| CEA$^+$ and/or CA 242$^+$ | 47 | 46 | 60 | 85 | 58 |
| CEA$^-$ CA 242$^-$ | 53 | 54 | 40 | 15 | 42 |

**Figure 1** Preoperative serum CEA levels of patients with colorectal cancer and patients with benign colorectal diseases. The dotted line represents the cut-off level of 5 ng ml$^{-1}$. The median values are shown by a line in each column.

**Figure 2** Preoperative serum CA 242 levels of patients with colorectal cancer and patients with benign colorectal diseases. The dotted line represents the cut-off level of 20 U ml$^{-1}$. The median values are shown by a line in each column.

**Figure 3** Receiver operating characteristic (ROC) curve analysis of CEA and CA 242 in colorectal diseases. A total of 260 patients with colorectal cancer were used for calculating the true-positive fraction and 92 patients with benign colorectal diseases were used for calculating the false-positive fraction. The true-positive fraction (TP) reflects the sensitivity of the test (sensitivity = TP × 100%), and the false-positive fraction (FP) the specificity [specificity = (1 − FP) × 100%]. The dotted line shows the 95% specificity level (FP = 0.05).

In Dukes A colorectal cancer 21% (eight patients) had an elevated CEA level only, 21% (eight patients) an elevated CA 242 level only, while 5% (two patients) had a concomitant rise in CEA and CA 242. Both markers were normal in 53% (21 patients) (Table IV, Figure 4).

In Dukes B cancer, 20% (20 patients) had an abnormal CEA alone, 14% (14 patients) an abnormal CA 242 alone, while 12% (12 patients) had both an elevated CEA and CA 242 level. A normal CEA and CA 242 serum level was seen in 54% (54 patients) of the patients (Table IV, Figure 4).

In Dukes C cancer 20% (12 patients) had an abnormal CEA alone, 22% (13 patients) an abnormal CA 242 alone and 18% (11 patients) had a concomitant rise of CEA and CA 242. Both markers were normal in 40% of the patients (Table IV, Figure 4).

In Dukes D cancer 18% (11 patients) had an elevated CEA alone, 8% (five patients) an elevated CA 242 alone, and 59% (36 patients) both an elevated CEA and CA 242 level. A normal CEA and CA 242 serum level was seen in 15% (nine patients) of the patients (Table IV, Figure 4).

When combining CEA and CA 242, requiring either or both to be elevated, the sensitivities were 47%, 46%, 60% and 85% in patients with Dukes A, B, C and D colorectal cancer respectively (Table IV) (Figure 4). The combination

**Figure 4** Correlation between serum CEA and CA 242 in patients with Dukes A–D colorectal cancer. Dotted lines at 5 ng ml$^{-1}$ for CEA and at 20 U ml$^{-1}$ for CA 242 divide the graph into four areas: patients with an elevated CEA serum level only, patients with an elevated CA 242 serum level only, patients with both markers elevated and patients with neither marker elevated. The correlation between CEA and CA 242 ($r^2$ values) is marked in each figure. The proportion of patients in each area is marked in the graph.

resulted in a significantly higher overall sensitivity of 58% ($P<0.001$) compared with either marker alone. The specificity decreased to 80%.

### Discussion

CEA is the most widely used tumour marker for colorectal cancer, although its value in primary diagnosis of colorectal cancer is questionable (Roberts, 1988; Kuusela *et al.*, 1991; Roberts *et al.*, 1992; Nilsson *et al.*, 1992). A sensitive and specific tumour marker for early diagnosis of colorectal cancer would be of great clinical importance, but none of the markers available today is sensitive and specific enough. The sensitivity of CA 19-9 and CA 50 has been shown to be clearly lower in all stages of colorectal cancer than that of CEA (Roberts, 1988; Kuusela *et al.*, 1991; Nilsson *et al.*, 1992). Concomitant use of CEA and CA 19-9 or CA 50, requiring either or both markers to be elevated, does not raise the sensitivity markedly compared with CEA alone (Kuusela *et al.*, 1987; Roberts, 1988).

Preliminary results on the new tumour marker CA 242 have been promising in patients with colorectal cancer (Kuusela *et al.*, 1991; Nilsson *et al.*, 1992). Although CA 242 is closely related to CA 19-9 and CA 50, CA 242 has shown clearly higher sensitivity for colorectal cancer than CA 19-9 and CA 50 (Kuusela *et al.*, 1991; Roberts *et al.*, 1992; Nilsson *et al.*, 1992). Therefore, a study comparing the preoperative serum levels of CA 242 with those of CEA was designed. The sensitivities of both markers proved to be of the same magnitude in all stage groups. However, there was no correlation between the serum values of CEA and CA 242, and mostly CEA and CA 242 were elevated in different patients. Only in Dukes D cancer was there a considerable overlap between the markers, but still without any correlation. These results are in concordance with our previously reported preliminary results and with those of Nilsson *et al.* (Kuusela *et al.*, 1991; Roberts *et al.*, 1992; Nilsson *et al.*, 1992). The low correlation between the markers indicates that CEA and CA 242 are expressed independently, which further supports the use of these markers concomitantly.

In calculating the specificities and predictive values, we used a control group consisting of patients with benign colorectal diseases, i.e. diseases relevant for differential diagnosis of colorectal cancer. Note that the proportion of elevated CEA and CA 242 values was of the same magnitude as that of healthy blood donors.

At the 90% specificity level the overall sensitivity was higher for CEA than for CA 242. However, when raising the specificity level to 95%, the sensitivity of CEA was clearly reduced, whereas the sensitivity of CA 242 remained unchanged and was superior to that of CEA. Combining two markers always results in reduced specificity. Combining CEA and CA 242 causes a 7–10% loss in specificity to 80%. This must be regarded as acceptable when compared with the 15–19% increase in sensitivity to 58%.

In conclusion, this study supports the concomitant use of CEA and CA 242 in the diagnosis of patients with colorectal cancer. A combination of CEA and CA 242 increased the sensitivity in all stages of colorectal cancer, particularly in Dukes A–C. A clinically even more important issue is whether the combination of CEA and CA 242 will show a similar increase in sensitivity also for recurrent disease. According to recently published results from Hall *et al.* (1994), the combination of CEA and CA 242 seems promising also in follow-up of patients with colorectal cancer, and this issue will also be evaluated in our patients in an ongoing study.

**Acknowledgements**
The authors thank Wallac Oy for kindly supplying the CA 242 test kits assay. This study has been supported by grants from Finska Läkaresällskapet and from Stiftelsen Dorothea Olivia, Karl Walter and Jarl Walter Perkléns minne and from Medicinska Understödsföreningen Liv och Hälsa.

### References

BRUMMENDORF T, ANDERER FA, STAAB HJ, HORNUNG A, STUMPF E AND KIENINGER G. (1985). Prognostic value of preoperative serum CEA level compared to clinical staging: III. An approach to scoring of prognostic factors in colorectal cancer. *J. Surg. Oncol.*, **28**, 263–269.

DEL VILLANO BC, BRENNAN S, BROCK P, BUCHER C, LIU V, MCCLURE M, RAKE B, SPACE S, WESTRICK B, SCHOEMAKER H AND ZURAWSKI VR. (1983). Radioimmunometric assay for a monoclonal antibody-defined tumor marker, CA 19-9. *Clin. Chem.*, **29**, 549–552.

GOLD P AND FREEDMAN SO. (1965). Demonstration of tumor-specific antigens in human colonic carcinomata by immunological tolerance and adsorption techniques. *J. Exp. Med.*, **121**, 439.

HAGLUND C, ROBERTS PJ, JALANKO H AND KUUSELA P. (1992). Tumour markers CA 19-9 and CA 50 in digestive tract malignancies. *Scand. J. Gastroenterol.*, **27**, 169–174.

HAGLUND C, LUNDIN J, KUUSELA P AND ROBERTS PJ. (1994). CA 242 – a new tumour marker for pancreatic cancer. *Br. J. Cancer*, **70**, 487–492.

HALL NR, FINAN PJ, STEPHENSON BM, PURVES DA AND COOPER EH. (1994). The role of CA-242 and CEA in surveillance following curative resection for colorectal cancer. *Br. J. Cancer*, **70**, 549–553.

HOLMGREN J, LINDHOLM L, PERSSON B, LAGERGÅRD T, NILSSON O, SVENNERHOLM L, RUDENSTAM C-M, UNSGAARD B, YNGVASON F, PETTERSSON S AND KILLANDER AF. (1984). Detection by monoclonal antibody of carbohydrate antigen CA 50 in serum of patients with carcinoma. *Br. Med. J.*, **288**, 1479–1482.

JOHANSSON C, NILSSON O, BÄCKSTRÖM D, JANSSON E-L AND LINDHOLM L. (1991*a*). Novel epitopes on the CA50-carrying antigen: chemical and immunochemical studies. *Tumor Biol.*, **12**, 159–179.

JOHANSSON C, NILSSON O AND LINDHOLM L. (1991*b*). Comparison of serological expression of different epitopes on the CA50 carrying antigen CanAg. *Int. J. Cancer*, **48**, 757–763.

KUUSELA P, HAGLUND C, ROBERTS PJ AND JALANKO H. (1987). Comparison of CA 50, a new tumour marker, with carcinoembryonic antigen (CEA) and alpha-fetoprotein (AFP) in patients with gastrointestinal diseases. *Br. J. Cancer*, **55**, 673–676.

KUUSELA P, HAGLUND C AND ROBERTS PJ. (1991). Comparison of a new tumour marker CA 242 with CA 19-9, CA 50 and carcinoembryonic antigen (CEA) in digestive tract diseases. *Br. J. Cancer*, **63**, 636–640.

LINDHOLM L, JOHANSSON C, JANSSON E-L, HALLBERG C AND NILSSON O. (1985). An immunometric assay (IRMA) for the CA 50 antigen. In *Tumour Marker Antigens*, Holmgren J (ed.) pp. 122–133. Studentlitteratur: Lund.

METZ CE. (1978). Basic principles of ROC-analysis. *Semin. Nucl. Med.*, **8**, 283–298.

MINTON J AND CHEVINSKY AH. (1989). CEA directed second-look surgery for colon and rectal cancer. *Ann. Chir. Gynaecol.*, **78**, 32–37.

NILSSON O, JANSSON EL, JOHANSSON C, LINDHOLM L. (1988). CA 242, a novel tumour associated carbohydrate antigen with increased tumour specificity and sensitivity. *J. Tumour Marker Oncol.*, **3**, 314.

NILSSON O, JOHANSSON C, GLIMELIUS B, PERSSON B, NORGAARD-PEDERSEN B, ANDRÉN-SANDBERG Å AND LINDHOLM L. (1992). Sensitivity and specificity of CA242 in gastrointestinal cancer. A comparison with CEA, CA50 and CA19-9. *Br. J. Cancer*, **65**, 215–221.

ROBERTS PJ. (1988). Tumour markers in colorectal cancer. *Scand. J. Gastroenterol.*, **23**, 50–58.

ROBERTS PJ, KUUSELA P, CARPELAN-HOLMSTRÖM M AND HAGLUND C. (1992). Value of different tumour markers in colorectal cancer. In *Tumor Associated Antigens, Oncogens, Receptors, Cytokines in Tumor Diagnosis and Therapy at the Beginning of the Nineties*, Klapdor RW (ed.) pp. 30–32. Zuckschwerdt, Munich.

RÖTHLIN MA, JOLLER H AND LARGIADÈ, RF. (1993). CA242 is a new tumor marker for pancreatic cancer. *Cancer*, **71**, 701–707.

TURNBALL RB, KYLE K, WATSON FR AND SPRATT J. (1967). Cancer of the colon: The influence of the no-touch isolation technic on survival rates. *Ann. Surg.*, **166**, 420–427.